# Identification and Characterization of a Mef2 Transcriptional Activator in Schistosome Parasites

**DOI:** 10.1371/journal.pntd.0001443

**Published:** 2012-01-03

**Authors:** John N. Milligan, Emmitt R. Jolly

**Affiliations:** 1 Department of Biology, Case Western Reserve University, Cleveland, Ohio, United States of America; 2 Center for Global Health and Disease, Case Western Reserve University, Cleveland, Ohio, United States of America; Queen's University Belfast, United Kingdom

## Abstract

Myocyte enhancer factor 2 protein (Mef2) is an evolutionarily conserved activator of transcription that is critical to induce and control complex processes in myogenesis and neurogenesis in vertebrates and insects, and osteogenesis in vertebrates. In Drosophila, Mef2 null mutants are unable to produce differentiated muscle cells, and in vertebrates, Mef2 mutants are embryonic lethal. Schistosome worms are responsible for over 200 million cases of schistosomiasis globally, but little is known about early development of schistosome parasites after infecting a vertebrate host. Understanding basic schistosome development could be crucial to delineating potential drug targets. Here, we identify and characterize Mef2 from the schistosome worm *Schistosoma mansoni* (SmMef2). We initially identified SmMef2 as a homolog to the yeast Mef2 homolog, Resistance to Lethality of MKK1P386 overexpression (Rlm1), and we show that SmMef2 is homologous to conserved Mef2 family proteins. Using a genetics approach, we demonstrate that SmMef2 is a transactivator that can induce transcription of four separate heterologous reporter genes by yeast one-hybrid analysis. We also show that Mef2 is expressed during several stages of schistosome development by quantitative PCR and that it can bind to conserved Mef2 DNA consensus binding sequences.

## Author Summary

Schistosome parasites infect more than 200 million people worldwide and cause human schistosomiasis. Free-swimming schistosome larvae are highly mobile and invade and penetrate the host's skin to perpetuate their lifecycle in their human host, growing from 90–215 micrometers in length as a schistosomulum to a 7–20 millimeter long adult worm. Few molecular pathways have been identified in schistosome worms that are important for parasite early development. The myocyte enhancer factor protein 2 is a major regulator of muscle and nerve development in mammals and insects and is highly conserved from bread yeast to vertebrates. Here we identify and characterize the Mef2 activator from parasitic schistosome worms, the first described in any parasitic worm, and delineation of its function may be important to further understanding the basic biology of schistosome early development. Additionally, since schistosomes developed early evolutionarily, an investigation of schistosome Mef2 regulatory mechanisms could lead to a greater understanding of the development of early muscle and neurogenic development in animals.

## Introduction

Schistosomiasis is a parasitic disease infecting over 200 million people worldwide with an at risk population of over 750 million [1].The disease is caused by blood fluke worms of the genus *Schistosoma*, primarily by the species *S. mansoni*, *S. haematobium*, and *S. japonicum*. Ninety-three percent of worldwide cases of schistosomiasis (192 million) occur in sub-Saharan Africa, with the highest prevalence of disease occurring in school aged children, adolescents, and young adults, where morbidity is causative of a variety of symptoms ranging from impaired physical growth to more than 280,000 deaths per year [2], [3], [4]. Due to the primary use of a single drug for treatment, praziquantel, concerns have arisen regarding the possible development of drug resistance, for which some reports suggest may be selected for in a laboratory setting [5]. Because of the profound global impact and the implications of schistosome caused disease, a complete understanding of the biology of these organisms is of importance to the research and medical community.

Schistosomes have a complex parasitic life cycle requiring molluscan and mammalian hosts and an intricate process of morphological and functional changes. Free-swimming cercariae infect the mammalian host via direct penetration of the skin. Upon host skin invasion, the small 90–215 micrometer long schistosomulum, develops a digestive tract, exchanges its glycocalyx for a tegument and must grow into a 7–20 millimeter long, muscular adult worm that is uniquely adapted to its host [6], [7], [8], [9], [10], [11], [12]. Following migration to the venules of the hepatic portal system, male and female worms pair and produce eggs, which are excreted from the host. Eggs hatch into miracidia, another morphologically distinct free-swimming stage that will infect the molluscan host. Within the snail, sporocysts develop and produce cercariae, which exit to restart the life cycle. Recent advancements have been made in schistosome research, including RNAi gene silencing studies [13], [14], microarray analyses [15], [16], proteomic analyses [17], [18], laser micro-dissection microscopy of tissues [19], and cell-specific labeling techniques allowing for in-depth tissue visualization [20]. While promising, there is still little known about the function and expression of genes within the schistosome parasite throughout its complex life cycle.

Myocyte enhancer factor (Mef2) proteins are members of the evolutionarily conserved MADS-box family, named after the four initially discovered members (Mini Chromosome Maintenance 1, AGAMOUS, Deficiens, and Serum Response Factor), found in yeast, *Arabidopsis thaliana*, *Antirrhinum majus*, and humans, respectively [21], [22], [23], [24]. The function and expression of Mef2 proteins have been investigated in many organisms, where they function as transcription factors that regulate cellular differentiation, morphogenesis, proliferation, T-cell selection, and survival [25], [26], [27]. Mef2 proteins also have a conserved Mef2 protein domain, which functions with the MADS domain in protein dimerization, DNA binding, and co-factor interactions; these domains are coupled with a highly variable, usually C-terminal domain, that functions in transcriptional activation [26], [28], [29]. Mef2 proteins bind as both homo- and heterodimers to a ten base pair Mef2 consensus sequence CTAWWWWTAG that is evolutionarily conserved across multiple species, although some variations to this consensus have been observed [30], [31], [32], [33].

The single Mef2 gene in *Drosophila* (D-Mef2) is perhaps the most studied Mef2 homolog [28], and is required for embryonic myogenesis [34]. Knockout studies have shown that the loss of D-Mef2 results in a complete loss of all muscle tissues [35] and loss of differentiation in all muscle cell lineages: somatic, cardiac, and visceral [36]. Chromatin immunoprecipitation (ChIP) and DNA microarrays have been used to elicit over 200 directly targeted genes in *Drosophila* and more than 650 regions in the genome bound by the activator [36], [37]. One such target, Actin57B, is directly regulated by D-Mef2 for differential expression in cardiac, skeletal, and muscle cell lineages, where it binds to the target consensus sequence CTATTTTTAG contained in the Actin57B promoter [38].

The vertebrate Mef2 family has four alternate spliceforms, Mef2A-D, characterized by highly varied C terminal activation domains [26] and overlapping, yet distinct patterns of expression [28]. In mammals, Mef2 requires interaction with other myogenic basic Helix Loop Helix transcriptional activators to direct myogenic differentiation [39]. The four homologs play a significant role in vertebrate heart development, where they act as regulators of other important cardiac transcription factors [28], [40]. In addition to its role in myogenesis, vertebrate Mef2 acts as a regulator of neural crest and craniofacial development in both zebrafish [41] and mammals [42], and aids in activation of bone development [43], neuronal differentiation [44], [45], muscle regeneration [46], and T-Cell development [28].

Mef2 proteins have conserved DNA binding sites. The vertebrate Mef2A, -C, and –D have similar DNA binding ability, although Mef2B exhibits a reduced binding efficacy [26]. Mef2A and Mef2D bind the most common Mef2 binding consensus sequence, CTAAAAATAG [33], [47]. The Mef2 homolog Rlm1, from the budding yeast, *Saccharomyces cerevisiae*, also binds this consensus sequence and can heterodimerize with mammalian Mef2A. Rlm1 functions in the mitogen activated protein kinase pathway as an important mediator in cell wall biosynthesis [48]. Recently, a Mef2 homolog in the liverwort plant species *Marchantia polymorpha (M. polymorpha)* was shown to play a role in gametophytic generation with affinity as a homodimer for the sequences CTATTTTTAG and CTATATATAG [49], showing the evolutionary conservation of Mef2 proteins and Mef2 binding properties.

This evidence suggests that Mef2 is a pivotal and highly conserved transcriptional activator, making it a prime target of interest in the functional genetics of *S. mansoni*. Here, we identify and characterize the *Schistosoma mansoni* Mef2 (SmMef2). We demonstrate that it is a functional transcriptional activator, that it is expressed during different stages of schistosome development, and that it recognizes Mef2 specific target sequences. Finally, we present several genes that may be potential downstream targets of SmMef2.

## Materials and Methods

### Bioinformatics

The protein sequence of the yeast Mef2 homolog Rlm1 was used for a Basic Local Alignment Tool for proteins (BLASTp) analysis against the *S. mansoni* genome databases GeneDB [50] and SchistoDB [51]. Identified sequences were then queried using Washington University protein Blast (WU-BLASTp) against the *S. cerevisiae* and *H. sapiens* genomes to compare MADS-box and Mef2 regions of homology. BLASTp analysis was done using the identified Mef2 homolog in *S. mansoni* against all genomes using NCBI BLAST (http://blast.ncbi.nlm.nih.gov) to identify other homologs across a variety of species. Identified protein sequences were downloaded and compared to *S. mansoni* Mef2 utilizing ClustalW2 (http://www.ebi.ac.uk/Tools/msa/clustalw2/) [52], [53]. The phylogram phylogenetic tree was drawn from a ClustalW-generated multiple sequence alignment of Mef2 homologs using the neighbor-joining method, and a Gonnet protein weight matrix with the gap open set at 10, the gap extension set at 0.2 and the gap distances set at 5.

### RNA Purification

Total RNA was purified from six-week old adult worms, four- hour schistosomula, cercariae, and sporocysts using Trizol Reagent purification (Invitrogen, Carlsbad, CA) and Purelink columns (Invitrogen, Carlsbad, CA). RNA was quantitated using a NANODROP 8000 spectrophotometer (Thermo Scientific, Waltham, MA) and gel analysis and RNA quality was assessed by visualization on a 2% agarose gel.

### Sample Preparation

Snails containing the Puerto Rican strain of *Schistosoma mansoni* originated from stocks maintained by the NIAID Schistosome Resource Center at the Biomedical Resource Institute (Rockville, MD). Transformation of cercariae and culturing of schistosomula were performed as previously described [54], [55].

### Molecular Cloning

DNA Primers using the InFusion Cloning System (Clontech, Mountainview, CA) were designed based on the identified Smp_129430 (SmMef2) spliced gene sequence and the pGBKT7 vector (Clontech, Mountainview, CA) following manufacturer's recommendations. Primers were ordered from Integrated DNA Technologies (IDT, Coralville, IA). Forward primer oAT007 (5′-GAA TTC CCG GGG ATC CGT CGA CTT ATG GGT CGC AAA AAA ATA CTC ATC AAG AAG-3′) and reverse primer oAT008 (5′-ATG CGG CCG CTG CAG GTC GAC TCA AAG GTG GCG CAC ACG TTT AAG AGG GTT-3′) were used to clone SmMef2 by the one-step RT-PCR SuperScriptIII/PlatinumTaq system (Invitrogen, Carlsbad, CA) using mixed total RNA from sporocyst, cercariae, and adult worms. The cDNA product was subcloned into vector pGBKT7 (Clontech, Mountainview, CA) at the Sal I site and this plasmid was used to transform chemically competent One Shot TOP10 cells (Invitrogen, Carlsbad, CA). Colonies were selected and grown in LB containing kanamycin liquid media. Plasmid DNA was purified using the Nucleospin Plasmid miniprep kit (Clontech, Mountainview, CA) and verified by restriction analysis and DNA sequencing to make plasmid pEJ1108.

SmMef2 from plasmid pEJ1108 was amplified by PCR and subcloned between Not I and Sal I sites of the expression vector pMAL-c5x (New England Biolabs, Ipswitch, MA) using InFusion (Clontech, Mountainview, CA) with forward primer oAT019 (5′-TCC ATG GGC GGC CGC ATG GGT CGC AAA AAA ATA CTC ATC AAG) and reverse primer oAT020 (5′-TTC GGA TCC GTC GAC TCA AAG GTG GCG CAC ACG TTT AAG AGG) to make plasmid pEJ1114. The product was analyzed by restriction analysis and sequenced.

### Modified Yeast 1-Hybrid System

Plasmid pEJ1108 was used to transform yeast strain AH109 (ordered from Clontech, Mountainview, CA; genotype *MATa, trp1-901, leu2-3, 112, ura3-52, his3-200, gal4Δ, gal80Δ, LYS2::GAL1_UAS_-GAL1_TATA_-HIS3, GAL2_UAS_GAL2_TATA_-ADE2,URA3::MEL1_UAS_-MEL1_TATA_-LacZ, MEL1*) and grown on SD -Trp plates. Transcriptional activity was tested using four different reporter genes present in the AH109 strain. Each reporter gene (*HIS3*, *ADE2*, lacZ, *MEL1;* encoding histidine 3, adenine 2, beta-galactosidase, and alpha galactosidase, respectively) is under control of a Galactose 4 protein (*Gal4*) dependent promoter and grown on selective synthetic media (SD). AH109 transformed with the pGBKT7 vector alone served as a negative control, and positive controls used AH109 transformed with pEJ780, a pGBKT7-based plasmid containing the full Galactose 4 transcript (GAL4). Expression of Histidine and Adenine auxotrophy was also tested by spot test analysis using four, 10-fold serial dilutions of liquid synthetic media without the amino acid tryptophan (SD –Trp), then grown on selective media missing either adenine, histidine or tryptophan (SD –Ade, SD –His, SD –Trp, respectively). O.D._600_ values of the positive control, negative control, and experimental cultures were matched before plating using the Nanodrop 8000. Growth was indicative of a positive result. Expression of MEL1 was tested by an α-galactosidase assay on SD -Trp where yeast cells were screened for blue color. Expression of lacZ was tested by a β-galactosidase assay and screened for blue color.

### Reverse Transcription and Quantitative PCR

RNA reverse transcription reactions were carried out using SuperScript III RT, RNAse OUT, and oligo (dT)_12–18_ (Invitrogen, Carlsbad, CA) using 1 µg of total RNA extracted from sporocysts, cercariae, schistosomula, and adult worms, as per manufacturer's recommendations. The reaction was carried out for one hour at 50°C, and treated with 10 U RNase H (New England Biolabs, Ipswitch, MA) and placed at 37°C for 20 minutes to remove any hybridized mRNA.

Quantitative Polymerase Chain Reaction (quantitative PCR) primers were designed using primer 3 software [56] (Table S1). Primers were checked for specificity using NCBI Primer-BLAST (http://www.ncbi.nlm.nih.gov/tools/primer-blast/) and for hetero- and homo-dimers and hairpin structures using Oligo Analyzer (http://www.idtdna.com/analyzer/applications/oligoanalyzer/). Quantitative PCR was performed using SYBR Green master mix on a StepOnePlus Real-Time PCR system with StepOne Version 2.0 software (Applied Biosystems, Carlsbad, CA), using 2 µL of the Reverse Transcriptase (RT) reactions described above. The following quantitative PCR conditions were used: 95°C for 10 minutes, 40 cycles of 95°C for 15 seconds and 60°C for 1 minute, followed by melt curve analyses. Replicates were manually screened, and those containing significant multiple melting peaks or bad passive reference signals were removed from analysis. Threshold cycle values and standard curve parameters were determined as described above. Cycle threshold (CT) values of Mef2 and potential downstream targets were evaluated by 2^−ΔΔCT^ methods using cyclophilin as a reference gene with sporocysts as an endogenous control. CT values for cyclophilin were consistent across all stages tested. Bar graphs were generated using StepOne software (Applied Biosystems). Error bars represent standard error [57].

### Protein Expression and Purification

Vectors pEJ1114 and pMAL-c5x (New England Biolabs, Ipswich, MA) that express either a Maltose binding protein fused to SmMef2 (MBP-SmMef2) or Maltose Binding Protein (MBP) alone were used to transform BL21 DE3 cells (Invitrogen, Carlsbad, CA). Cultures were shaken at 37°C to an O.D._600_ of 0.9 and protein expression was induced with IPTG at a final concentration of 2 mM. The culture was shaken for 19.5 hr at 20°C. Cell extracts were prepared as outlined in the pMAL Protein Fusion and Purification System manual (New England Biolabs, Ipswitch, MA). Briefly, cells were resuspended in column buffer with PMSF and Halt protease inhibitor cocktail (Thermo Scientific, Waltham, MA), lysed with lysozyme and pulse sonification, and cleared by centrifugation at 12,500×g for 30 min. Protein was purified by diluting cell extract 1∶6 and running through a gravity column consisting of 10 mL Amylose High Flow Resin (New England Biolabs, Ipswitch, MA). Protein was eluted with maltose and then transferred to storage buffer by utilizing 100,000 MWCO Amicon Ultra 2 ml centrifugal filters. Total protein concentration was quantitated by Bradford assay.

### Electrophoretic Mobility Shift Analysis

DNA binding oligonucleotide sequences were designed based on the Actin57B promoter region from *Drosophila melanogaster*, specifically, the region from −218 bp to −188 bp of the translation start codon (5′-GCTGAAGGAT{ctatttttag}GCGGATCGGC-3′), which contains a *Drosophila* Mef2 binding site (internal brackets) [38]. Three double-stranded oligonucleotide pairs AT11, AT12, and AT13 (labeled as F for forward and R for reverse complement) were designed that make three different versions of the Mef2 binding consensus. Oligonucleotide oAT11F (5′-GCTGAAGGAT{ctatttttag}GCGGATCGGC-3′), the forward sequence for the double stranded oligo-pair AT11, and reverse complement oligonucleotide oAT11R (5′- GCCGATCCGCCTAAAAATAGATCCTTCAGC-3′) were used. For subsequent oligos, the internal Mef2 site was modified to test other Mef2 binding sites. Double-stranded oligonucleotide (ds-oligo) AT12 contains one of the most common consensus binding sequences [47], [58], made up of the forward and reverse complement oligonucleotides oAT12F (5′-GCTGAAGGAT{ctaaaaatag}GCGGATCGGC-3′) and oligonucleotide oAT12R (5′-GCCGATCCGCCTATTTTTAGATCCTTCAGC-3′). Ds-oligo AT13 contains an *M. polymorpha* Mef2 binding site, generated with oligonucleotide oAT13F (5′-GCTGAAGGAT{ctatatatag}GCGGATCGGC-3′) and oAT13R (5′- GCCGATCCGCCTATATATAGATCCTTCAGC-3′) [49]. Ds-oligo AT14 contains the DNA binding site for the *S. cerevisiae* gene *NDT80* as a negative control, made with oAT14F (5′-GCTGAAGGAT{gtcacaaaat}GCGGATCGGC-3′) and oAT14R (5′- GCCGATCCGCATTTTGTGACATCCTTCAGC-3′) [59], [60]. For all double-stranded “hot” oligonucleotides, the forward oligonucleotides were designed with a 5′ biotin label, while “cold” double-stranded oligonucleotides were designed with no biotin label; all oligonucleotides were ordered from Integrated DNA Technologies (IDT, Coralville, IA). Oligonucleotides were annealed by mixing equimolar amounts of forward and reverse oligonucleotides in .05 M NaCl, .01 M Tris pH8.0, and 1 mM EDTA. The oligonucleotides were boiled at 95°C for 10 min, and then cooled 1°C per 60 sec down to 23°C on a Multigene Thermal Cycler (Labnet Technologies, Edison, NJ).

Electrophoretic Mobility Shift Assay (EMSA) reactions were performed using the LightShift Chemiluminescent EMSA kit (Thermo Scientific, Waltham, MA) as described by the manufacturer. Briefly, binding reactions contained 180 fmol hot dsDNA, 36 pmol (200×) cold dsDNA (where applicable), 1.4 µg of Amylose-purified protein extract (MBP-SmMef2 or MBP), and 1× supplied binding buffer, 2.5% glycerol, 5 mM MgCl_2_, 50 ng/µL Poly (dI-dC) Inhibitor DNA, and .05% NP-40. Binding reactions were prepared on ice and placed at room temperature for 20 min. Reactions were mixed with supplied 5× loading buffer, and loaded onto a prerun 10 cm×10 cm 5% polyacrylamide/0.5× TBE native gel, and run in 0.5× TBE for 70 min at a constant voltage of 150V. The gel was placed in a blotting apparatus with a Biodyne B Pre-Cut Modified Nylon Membrane (size 0.45 µm, Thermo Scientific, Waltham, MA) and transferred in 0.5× TBE at constant current of 380 mA for 1 hr. Following transfer, the membrane was crosslinked on a CL-1000 Ultra Violet crosslinker (UVP, Upland, CA) on automatic settings for 120 mJ/cm^2^. The membrane was developed according to kit protocol, and visualized with a Charged Coupled Device (CCD) camera set to an exposure time of 1–2 min.

### Downstream Targets of SmMef2

Potential downstream targets of SmMef2 were screened for Mef2 binding sites within 5000 bp upstream of the expected translation start codon. Fifty-six *S. mansoni* genes were screened and their upstream sequences were obtained from GeneDB [50]. Putative targets were selected for screening based on suspected gene function or high levels of homology as revealed by BLASTp analysis against known Mef2 targets in *D. melanogaster* [36]. Two types of binding sites were used for screening; strong consensus binding sites were defined by the sequence CTAWWWWTAG, the Mef2 consensus sequence [30], [32], [33], while weak consensus binding sites were defined by either CTTWWWWTAG or CTAWWWWTAA. These sites differ from the common consensus by a single nucleotide in either the third or the last base pair position. The two “weak” sequences were selected based on a screening of over 200 Mef2 binding sites, which determined these two sequences to be the next most frequently occurring binding sites for Mef2 after the consensus sequence [33]. Binding sequences and distance upstream of the translation start codon were noted.

## Results and Discussion

### Schistosome Parasites Have a Mef2-Like Protein

BLASTp analysis with the yeast Mef2 homolog Rlm1 against the *Schistosoma mansoni* genome database identified a putative myocyte enhancer factor 2 (Smp_129430), which contains conserved MADS-box and Mef2 domains (Figure S1). A WU-BLASTp with the putative SmMef2 against the *S. cerevisiae* yeast genome showed that the schistosome Mef2 and the yeast Mef2 homolog, Rlm1 are 49% identical and 68% similar across 88 amino acids at the N-terminus, which encodes the MADS Box and Mef2 domains of putative SmMef2 and Rlm1 (Figure S1A). BLASTP against the human Mef2A protein showed greater similarity (Figure S1B). We observed that putative SmMef2 and human Mef2A, Mef2B, Mef2C and Mef2D proteins are 78–80% identical and 90% similar across amino acids 1–88 at the N-terminus (data not shown). These data agree with known homology between other Mef2 homologs, where the Mef2 and MADS-box domains, particularly the MADS-box, are highly conserved across species [26], [28]. When the remaining C-terminus (amino acids 89–661) of putative SmMef2 was used to search for homologs by BLASTp in NCBI, excluding putative SmMef2, we found no protein, nor protein domains with any significant homology. Although the sequence similarity of the DNA binding domains of putative SmMef2 and human Mef2 proteins A-D are highly conserved, the lack of conservation among the activation domain of SmMef2 is not unsubstantiated as the activation domain of Mef2 proteins characteristically displays a high degree of variation, even across homologs within the same species [26], [33].

### Schistosome Mef2 Is a Transcriptional Activator

**Figure 1:**
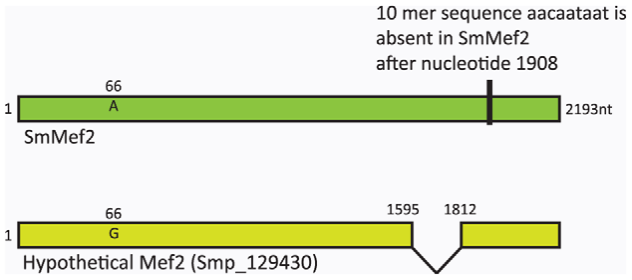
The SmMef2 expressed sequence is different from the published sequence, Smp_129430. The SmMef2 is 2193 nucleotides and encodes for a 731 amino acid protein. It differs from the currently published sequence the following ways: 1). A G to A transition occurs at nucleotide 66. This does not affect the amino acid sequence 2). A sequence from nucleotides 1596–1811 is recognized as an intron in the database, but this sequence is included in the functional coding sequence, and 3). There is a ten base pair deletion of nucleotides aacaataat in SmMef2 after nucleotide 1908.

To test whether putative SmMef2 is actively expressed, we extracted RNA from sporocysts, cercariae, and adult worms. Using a 1∶1∶1 mixture of RNA from these three developmental stages for reverse transcriptase PCR (RNA from mixed developmental stages permits amplification in fewer reactions when the expression profile of a gene is unknown), we cloned a 2,193 base pair sequence encoding 731 amino acids. The cloned sequence is 67 amino acids larger than predicted in the schistosome database for the putative Mef2 (Smp_129430). The extra 201 nucleotides are located in predicted intron 4. There is also a nine-nucleotide deletion AACAATAAT after nucleotide 1908 of SmMef2 (Figure 1). The corresponding nucleotide and protein sequences are found in Figure S2. The BLASTp analysis described above was repeated with the sequenced version of putative SmMef2 with similar results, which will be referred to as SmMef2, to distinguish it from sequence Smp_129430. The SmMef2 DNA sequence has been submitted to the Genbank database under accession number JN900476.

Mef2 proteins are proposed to have arisen out of a duplication of the ancestral MADS box domain before the divergence of plants and animals [61]. In plants, the Mef2-like sequences are characterized as type II MADS box proteins. To further characterize SmMef2 relative to other Mef2 proteins, we constructed a Mef2 phylogenetic tree using ClustalW that includes sequences from yeast, plants, amphibians, insects, and humans (Figure S3). The phylogenetic tree demonstrates that SmMef2 is linked to other Mef2 proteins. We find it intriguing that SmMef2 is classified within the same lineage as mammalian Mef2B, suggesting that it might be a Mef2B protein, although at this point we are not convinced there are enough data to support that argument. In concurrence with other studies, this phylogenetic tree suggests that Mef2B is divergent from other Mef2 proteins in mammals [62].

**Figure 2:**
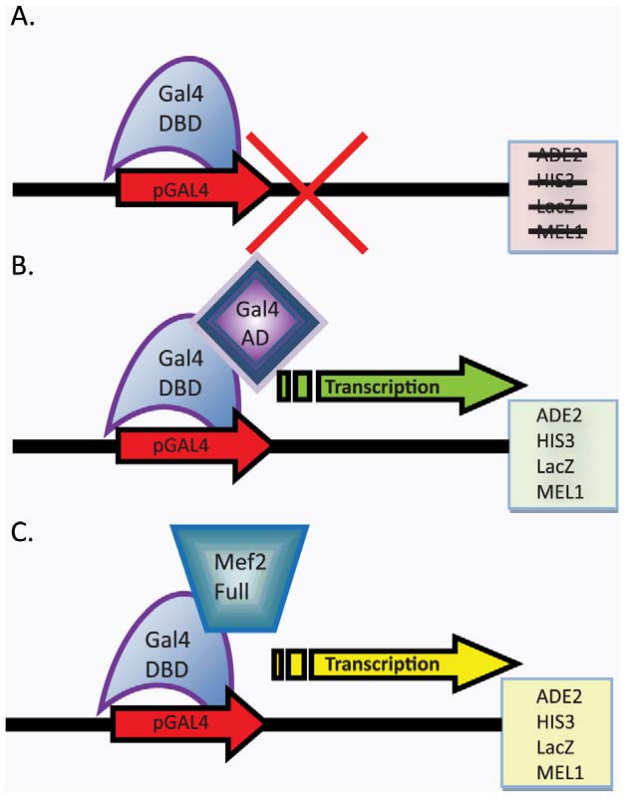
A graphical depiction of the modified yeast 1-hybrid system. The Gal4 DBD alone cannot activate reporter genes activity without an activation domain (A), while the complete Gal4 transcript will induce reporter activity (B). A fusion protein of Mef2 and the Gal4-DBD can activate the reporter if Mef2 is a transcriptional activator (C).

**Figure 3:**
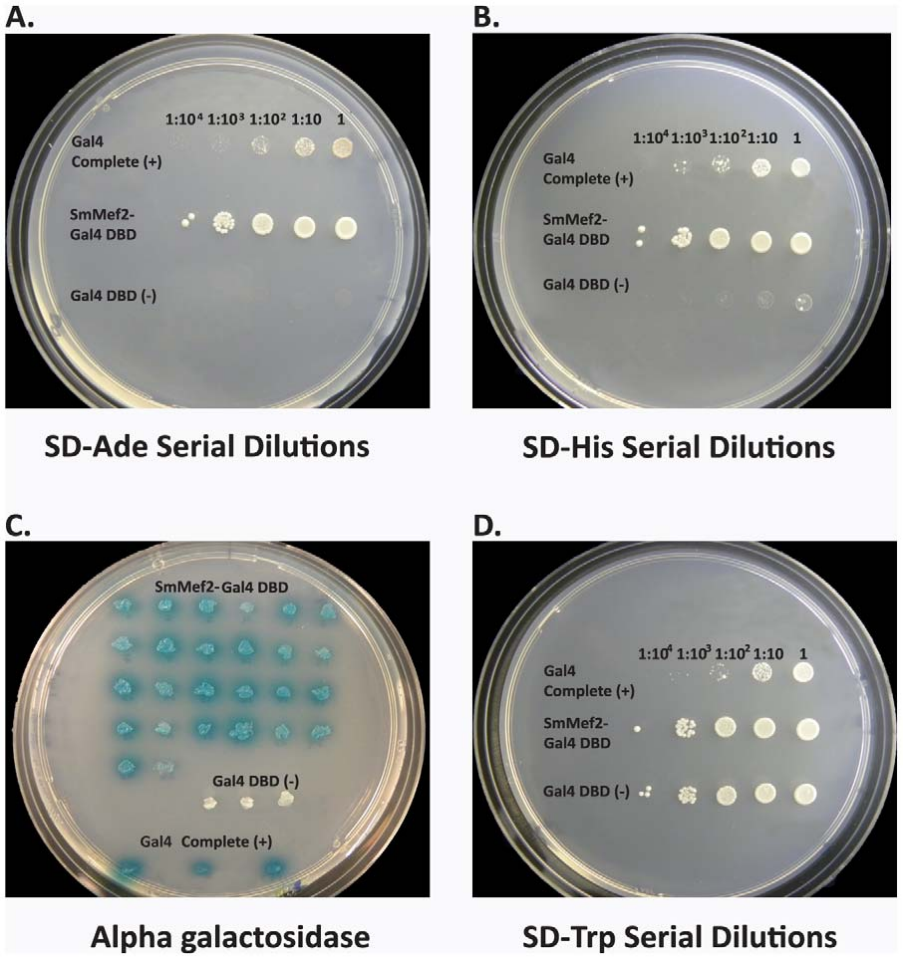
Selection and screening to test ability of SmMef2 to induce reporter genes. Serial dilution spot tests are displayed on –adenine or –histidine nutritional marker plates to test whether SmMef2 can induce the genes encoding adenine or histidine biosynthesis (A and B, respectively). Cells are diluted from right (undiluted) to left in 10 fold increments. Alpha galactosidase assay to test whether SmMef2 can induce the MEL1 reporter gene (C), where blue color indicates reporter gene activation. The negative control is white. To control for growth alone, cell are grown on media without tryptophan, which selects for the presence of the pGBKT7 plasmid (D).

Many Mef2 proteins encode for activators of transcription. If SmMef2 is a Mef2 protein, then it should potentially be able to function as a transcriptional activator (TA). To test whether SmMef2 can function as a TA, we took a yeast 1-hybrid genetics approach, utilizing the yeast expression plasmid pGBKT7 (Clontech, Mountainview, CA) and yeast strain AH109 (Clontech, Mountainview, CA). A fusion protein was generated combining the DNA binding domain of Gal4 (Gal4-DBD), from the expression plasmid pGBKT7, with the full-length SmMef2. The AH109 strain used for the 1-hybrid analysis contains yeast *GAL1* and *GAL2* promoters that controls expression of 4 different reporter genes, Histidine 3 (*HIS3)*, Adenine 2 (*ADE2)*, beta galactosidase (lacZ), and alpha galactosidase (*MEL1)*. The Gal4-DBD alone cannot activate the reporter genes and was used as a negative control (Figure 2A), whereas the complete Gal4 protein with it own activation domain can activate the reporter genes and was used as a positive control (Figure 2B). If SmMef2 is a TA, then when fused to the Gal4 DBD, it will drive transcription by utilization of the transactivation domain from SmMef2 (Figure 2C). To test the levels of reporter activity for *ADE2* and *HIS3* reporter genes, we performed standard spot tests at comparable dilutions on test plates with nutritional markers. All cells were grown to log phase, then diluted to equal cell counts measured to within 0.01 optical density 600 nm (OD_600_). These were then serially diluted and grown for 3 days at 30°C. We found that SmMef2 displayed transcriptional activity in all reporters tested (Figure 3). Serial dilutions of SmMef2 on SD -Ade and SD -His plates grew exceptionally well and did not show a reduction in spot size until 10^4^-fold dilution, whereas the positive control had a reduction of growth at a dilution factor of 10^2^ (Figure 3A, 3B). The negative control (Gal4-DBD alone) did not elicit auxotrophy for adenine or histidine (Figure 3A, 3B). Gal4 is a strong transcriptional activator in yeast, but we observed that cells expressing the positive control (full length Gal4 protein) did not grow as well as cells expressing Mef2 on SD –Ade or SD –His plates [63], [64]. This is not surprising as there is sufficient evidence suggesting that one transcriptional coactivator can interfere with the function of another. This has been reported when using overexpression of Gal4, where the activator is so strong that it deleteriously affects general transcription, resulting in reduced growth rate [65]. To directly test whether slower growth is due to a reduced ability of the positive control to activate the reporter genes relative to SmMef2, or whether it is a reduction in growth rate due to overexpression of the full length Gal4 protein, we tested growth on synthetic media without tryptophan. The plasmid, pGBKT7 carries a gene that encodes for tryptophan auxotropy, and was used to clone the Gal4-DBD/SmMef2 fusion protein and the full length Gal4 protein. Growth on synthetic media without tryptophan (SD –Trp) does not select for reporter activity but selects for the presence of the pGBKT7 plasmid; therefore, all cells should grow equally well. On media without tryptophan, the positive control expressing full length Gal4 protein grows slower than either Mef2 or the negative control, which has the plasmid expressing the Gal4-DBD alone (Figure 3D). The growth on SD –Trp correlates with the growth rate seen on SD –Ade and SD –His, and corroborates that the slower growth of the positive control is not due to an inability to activate the reporter genes.

SmMef2 was also tested for positive transcriptional activity using an alpha galactosidase assay. In this screen, cells expressing the *MEL1* reporter gene turn blue in the presence of X-alpha-gal (Clontech, Mountainview, CA), whereas colonies not expressing the *MEL1* reporter will remain white. Twenty-six transformants were picked, patched, and screened for reporter activity. All 26 colonies tested were blue (Figure 3C). The positive control was blue and the negative control was white, as expected (Figure 3C). Similar results were also observed when SmMef2 was tested using the beta galactosidase assay (data not shown). These data demonstrate that SmMef2 is a functional activator. The Gal4 system is a common method for testing transcriptional activity in eukaryotes via heterologous *in vivo* expression in yeast [64]. Our demonstration of this approach using a schistosome genes suggests that this approach could be a viable strategy to elucidate functional TAs in *S. mansoni*, as described previously in budding yeast [66].

### SmMef2 Binds Conserved Mef2 Consensus Sequences

**Figure 4:**
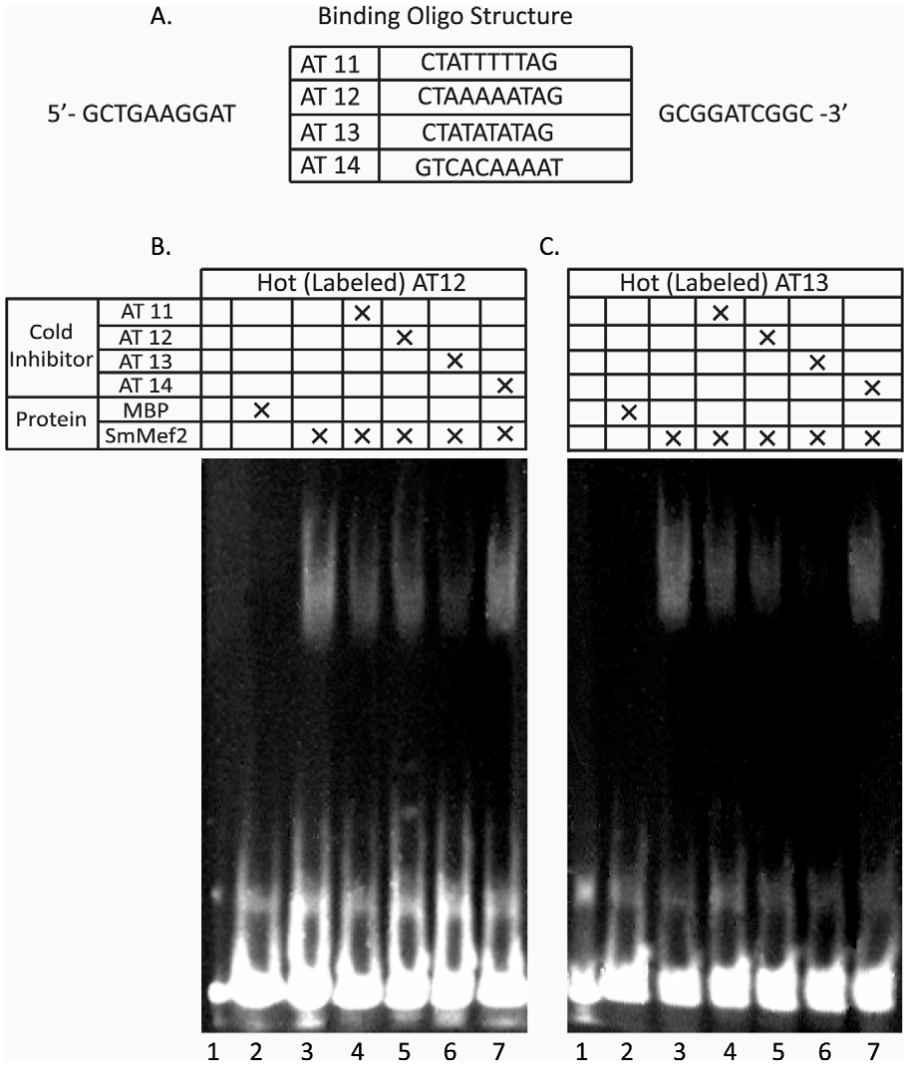
EMSA Analysis of SmMef2 DNA binding. Double stranded oligonucleotides with Mef2 consensus sequences were designed to test SmMef2 binding requirements (A). AT14 is the negative control probe (A). An MBP-SmMef2 fusion protein was purified and tested for its ability to bind double-stranded labeled probes (oligo-pair AT12 and oligo-pair AT13) containing different Mef2 consensus sequences (B and C, respectively). Purified MBP alone does not bind the DNA probes (B and C Lanes 2), while the MBP-SmMef2 fusion protein binds the probes (B and C, Lanes 3). Unlabeled oligonucleotide pairs AT11, AT12 and AT13 compete for binding against the labeled reporters (B and C, Lanes 4–6), while nonspecific AT14 does not compete (A and B Lane 7).

Mef2 proteins bind DNA directly to regulate diverse developmental programs. Mef2 proteins recognize the DNA consensus CTAWWWWTAG and bind either as a homo or heterodimer [28], [31], [67], [68]. We proposed that if SmMef2 is a Mef2 protein, that it should be able to recognize a version of the Mef2 DNA consensus. To address binding capabilities of the SmMef2, we made a protein hybrid of SmMef2 that is N-terminally fused to the maltose binding protein (MBP-SmMef2). The MBP-SmMef2 protein hybrid was expressed and purified from bacteria and tested by electrophoretic mobility shift assay (EMSA) to determine whether it could recognize Mef2 consensus sequences (Figure 4A). Forward (F) and Reverse (R) complementary DNA oligonucleotides were designed based on a 30-basepair region of the *Drosophila melanogaster* Actin 57B promoter which contains a centralized Mef2 DNA-binding site. We cloned three different variations of the centralized core Mef2 sequence CTAWWWWTAG (Figure 4A). Double-stranded oligonucleotide (ds-oligo) AT11 has the sequence CTATTTTTAG, the wildtype sequence found in the *Drosophila* Actin 57B promoter. Ds-oligo AT12 is the more commonly observed Mef2 consensus, CTAAAAATAG, and is recognized by human Mef2A and Mef2C proteins. The Mef2 consensus in ds-oligo AT13 (CTATATATAG), was identified in the liverwort *M. polymorpha* and is a perfect palindrome. Finally, as a negative control, ds-oligo AT14 GTCACAAAA, does not contain a Mef2 binding consensus; it is replaced with the middle sporulation element (MSE) that is recognized by the yeast Ndt80 protein, a meiosis-specific transcriptional activator [59], [60], and should not be recognized by SmMef2.

The EMSA data produce 3 bands; the lower band is single-stranded, biotin-labeled DNA and is consistent across all samples; the mid-lower band is double-stranded labeled DNA; and the upper band is DNA shifted due to protein binding by SmMef2. We find that SmMef2 can bind to all three Mef2 consensus ds-oligo sequences, AT11 (CTATTTTTAG, data not shown), AT12 (CTAAAAATAG), and AT13 (CTATATATAG); Figures 4B and 4C, Lanes 3). To address whether SmMef2 preferentially binds either consensus sequence, we tested whether unlabeled “cold” AT11, AT12, or AT13 could compete for binding against labeled probe AT12 or AT13. We initially predicted that SmMef2 would most likely prefer the mammalian consensus, but under these conditions, it preferred the consensus sequence from the liverwort *M. polymorpha*, and followed by (in order of preference) AT12 and AT11 (Figure 4B and 4C, Lanes 3–6). Ds-oligo AT14, the negative control, did not compete against labeled probe for binding (Figure 4B and 4C, Lanes 7). MBP protein alone was not able to bind AT11 (not shown), AT12 or AT13 (Figure 4B and 4C, Lanes 2), demonstrating that SmMef2 was solely responsible for the shift. These data, in combination with sequence conservation and the ability for the SmMef2 to act as a TA, provide strong evidence that SmMef2 encodes a Mef2 homolog. Therefore, we name this gene SmMef2 for *Schistosoma mansoni* myocyte enhancer factor 2.

### SmMef2 Developmental Expression

Mef2 proteins play significant roles in development, particularly in early myogenesis and neurogenesis. We asked when SmMef2 is expressed during schistosome development. To address this question, we extracted RNA from sporocysts, cercariae, 4-hour schistosomula, and adult worms and measured transcript levels by quantitative PCR. There is little literature focused on myogenesis or neurogenesis in schistosomes, although several electrophysiological studies on muscle and nerve cells or physiological descriptions have been published [69], [70], [71], [72], [73]. We made a simple prediction that these developmental stages might undergo myogenesis or neurogenesis for the following basic functions: 1) sporocysts- to produce fully developed and swimming cercariae that must exit the molluscan and follow chemical and visual cues to find and invade a mammalian host, 2) cercariae- for the reasons just mentioned, 3) schistosomula- to produce new muscle and neurons needed for motility, elongation, growth and development into adult worms after transformation, and 4) adult worms- for muscle motility, and muscle neuronal maintenance. There is some discussion that myogenesis in newly transformed schistosomula and adult worms might occur at the site of cercarial tail separation, proposed due to the characterization of cercarial and adult musculature patterns and the speculation that development of adult musculature occurs directly from larval musculature or undifferentiated stem cells derived from larvae [72]. This could suggest that if Mef2 is involved in myogenesis, its expression should be correlative with stages during which muscle development occurs.

**Figure 5:**
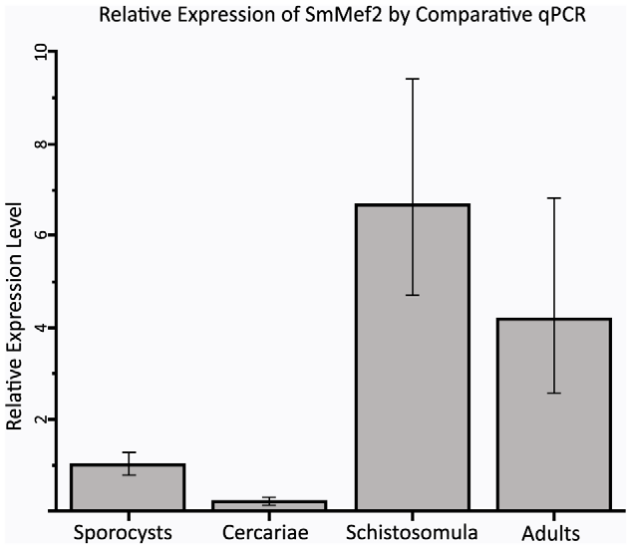
SmMef2 Expression during development. Quantitative PCR analysis of SmMef2 transcript profile during schistosome development was performed using cyclophilin as a reference control.

Quantitative PCR was used to generate an expression profile of Mef2, with cyclophilin used as a reference gene and the sporocyst stage used as a calibrator (Figure 5). Our data show a significant enrichment of SmMef2 beginning at the schistosomula stage, with decreased levels in the adult stage. The 6-fold increase in expression from sporocysts to schistosomula is especially striking given that levels drop 5-fold in cercariae; this means that Mef2 expression is upregulated over 30-fold during the transition from cercariae to schistosomula. If SmMef2 is involved in myogenesis, then this observation is consistent with the proposal by Mair *et al* suggesting that the origins of myogenesis initiate after larval transformation and continue in the adult worm [72], and this increase correlates with significant changes in the organism's morphology and musculature during its transition to an adult.

### Schistosomes Have Several Potential Mef2 Transcriptional Targets

Since schistosome parasites express Mef2, we asked whether we could bioinformatically identify potential targets of SmMef2. We screened for possible schistosome homologs of 56 genes regulated by Mef2 in *Drosophila* [36] using a simple BLASTp analysis. Twenty-six genes had either weak or strong Mef2 binding sequences within 5,000 bp upstream from the predicted translation start site, while 8 of these genes contained the core Mef2 consensus sequence CTAWWWWTAG (Table 1). Three of these genes (DNA replication licensing factor MCM7, actin, and four and a half LIM domains) contain a sequence tested by our EMSA analysis (Table 1). Sixteen genes are putatively involved in muscle development, three in neuronal development, and one gene in mini chromosome maintenance. Several proteins identified are homologous to those in *Drosophila*. These include: three actin genes homologous to Actin57B ; four tubulin beta chains homologous to the beta tubulin gene 60D (Btub60D); BMP antagonist noggin and Transforming Growth Factor beta (TGF-ß) family homologs to Drosophila TGFß homolog Decapentaplegic (Dpp); two Wnt-related proteins (Wnt proteins are involved in neurogenesis, patterning and development) [74], [75]; crp1/csrp1/crip1 and two, four and a half LIM domains proteins with homology to Muscle LIM protein at 60A (Mlp60A); and a Genomic Screened Homeobox (Gsx) family homeobox protein with homology to Mesenchyme homeobox 2 protein (Mox2), that is important for early mesoderm patterning [76] and limb muscle development [77] in mice, and which is correlative with SmMef2 being involved in myogenesis in schistosome parasites. Each of these genes has a Mef2 binding site, suggesting that they may be potential targets of SmMef2.

**Table 1:** Putative targets of SmMef2.

Putative gene type (name)	Smp number	Binding site sequence	Distance upstream	Gene ID (NCBI)
actin*	Smp_046590	cttttattaa	582	8349760
actin* #	Smp_077850	CTATTTTTAG	3565	8345287
actin*	Smp_161920	cttattatag, cttaatttag	2013, 3694	8349219
BMP antagonist noggin*	Smp_099440	ctaattataa	31	8351146
crp1/csrp1/crip1* #	Smp_087250	CTAAATATAG	1921	8355365
DNA replication licensing factor MCM7#	Smp_032500	CTAAAAATAG	455	8348621
four and a half LIM domains*	Smp_048560	ctataaataa, ctattaataa	2587, 4712	8341782
four and a half LIM domains* #	Smp_143130	ctttattag, cttttattag, cttaaaatag, CTAAAAATAG	169, 2013, 4436, 4713	8351888
GLUT4	Smp_050640	ctaaaaataa, ctttttatag	230, 600	8343690
gsx family homeobox protein*	Smp_138140	ctatttataa	1382	8347764
myosin XV	Smp_127510	ctattattaa	1785	8353008
netrin#	Smp_146840	CTAAATATAG, ctaatatta	1403, 4948	8348295
netrin	Smp_151310	ctaatattag, ctaattttaa	1782, 2823	8340778
semaphorin 5-related#	Smp_158550	CTAAATATAG	3459	8343948
TGF beta family*	Smp_063190	cttaaaatag, ctatatttaa	310, 1172	8347411
tropomyosin#	Smp_022170	CTAAATATAG	791	8343498
tropomyosin	Smp_044010	ctttaattag, ctttttttag	1403, 4077	8350309
tropomyosin#	Smp_085290	CTATTATTAG	1775	8347052
tubulin beta chain*	Smp_078040	ctaaaaataa	4193	8353635
tubulin beta chain*	Smp_079960	ctaaatttaa	1099	8347203
tubulin beta chain*	Smp_079970	ctatttttaa, ctaaatttaa	1777, 2811	8347204
tubulin beta chain*	Smp_035760	ctatttttaa	2832	8345905
tubulin delta chain	Smp_154880	cttaatttag	3246	8342529
tubulin epsilon chain#	Smp_028360	CTAATATTAG	948	8343061
WNT related* #	Smp_167140	ctataaataa, CTAATAATAG, ctttatttag	456, 1387, 4817	8352146
WNT related* #	Smp_152900	CTATAAATAG	433	8345780

**KEY.**

*:blasted from D. melanogaster gene with know Mef2 binding sites.

# : contains upstream sequence conforming to the strict Mef2 consensus.

Schistosome homologs of known Drosophila Mef2 transcriptional targets were individually screened for Mef2 binding sites within 5000 base pairs of the translation start site. Genes were screened for strong consensus (capital letters, highlighted in grey: CTAWWWWTAG) and weak consensus sequences (lower case letters: CTWWWWWTAG or CTAWWWWTAR). Upstream distances from the start codons are noted in base pairs.

Using quantitative PCR, we analyzed the transcriptional profile of three genes (Netrin- Smp_146840, tropomyosin- Smp_022170, and tubulin e-chain- Smp_028360) in sporocysts, cercariae, 4 h schistosomula, and adults. Each of these genes has potential Mef2 binding sites located within 1500 base pairs upstream of the translation start sites. Unfortunately, the initial results were inconclusive, although expression level seemed to increase in adults consistently (data not shown). One explanation for this result may be that the Mef2 gene is transcribed, but Mef2 protein has not yet activated its targets. This can be addressed by looking at transcript levels of potential SmMef2 targets at later time points.

### Conclusion

Here we show that schistosome parasites express a myocyte enhancer factor 2, which we name SmMef2 for *Schistosoma mansoni* myocyte enhancer factor 2. We identified SmMef2 in a search for schistosome homologs to yeast transcriptional activators. SmMef2 has the conserved MADS box and Mef2 domains found in Mef2 activators. Mef2 proteins play a role in transcriptional activation. Using the yeast 1-hybrid and EMSA, we show that SmMef2 is an activator of transcription and that it specifically recognizes Mef2 consensus sequences *in vitro*. Furthermore, quantitative PCR data show a developmentally regulated pattern of expression with comparatively high transcript levels in four-hour schistosomula and adult worms. This leads us to propose that SmMef2 may play a significant role in the development from schistosomula to adult worms. Finally, we describe potential targets of Mef2 genes that are found in schistosomes and contain consensus sequences in their promoter regions. Taken together, these data provide the first description of a Mef2 activator in a helminth. Mef2 resides at an early point in the evolution of animals. Understanding how myogenesis works in schistosomes could provide insights into the evolution of mammalian myogenesis. In addition, although we know that the MADS box and Mef2 domains are highly conserved, we observed that outside of this region, SmMef2 varies in sequence dramatically from its mammalian and insect homologs. This difference might provide an opportunity for its exploitation as a potential drug target.

## Supporting Information

Figure S1 **Mef2 Sequence Analysis is seen.** Sequence analysis of SmMef2 (Smp_129430) against the yeast gene Rlm1 (A) and human Mef2A (B). The conserved MADS-box (57 amino acids) and the Mef2 domains (29 amino acids) are labeled.
(TIF)

Figure S2 **SmMef2 DNA and Protein Sequences are demonstrated.** SmMef2 was cloned and sequenced. A G to A transition at nucleotide is underlined, italicized and in bold. An ** is placed after nucleotide 1908 where the deletion of the 10 nucleotides AACAATAAT occurs. The coding sequences between nucleotides 1595 and 1812, previously described as an intron, encode for protein. These sequences are underlined.
(TIF)

Figure S3 **Phylogenetic tree comparison of Mef2 homologs using ClustalW.** A phylogram phylogenetic tree was drawn from a ClustalW-generated multiple sequence alignment of Mef2 homologs using the neighbor-joining method (Gonnet protein weight matrix, with the gap open set at 10, the gap extension set at 0.2 and the gap distances set at 5).
(TIF)

Table S1 **Gene names and DNA primer sequences used for quantitative PCR analysis.**
(DOC)
