# Supplementary figures and images for: Identification and Characterization of a Mef2 Transcriptional Activator in Schistosome Parasites
Source: PLoS Negl Trop Dis. 2012 Jan 3;6(1):e1443. doi: 10.1371/journal.pntd.0001443 (PMC3250504; doi:10.1371/journal.pntd.0001443)

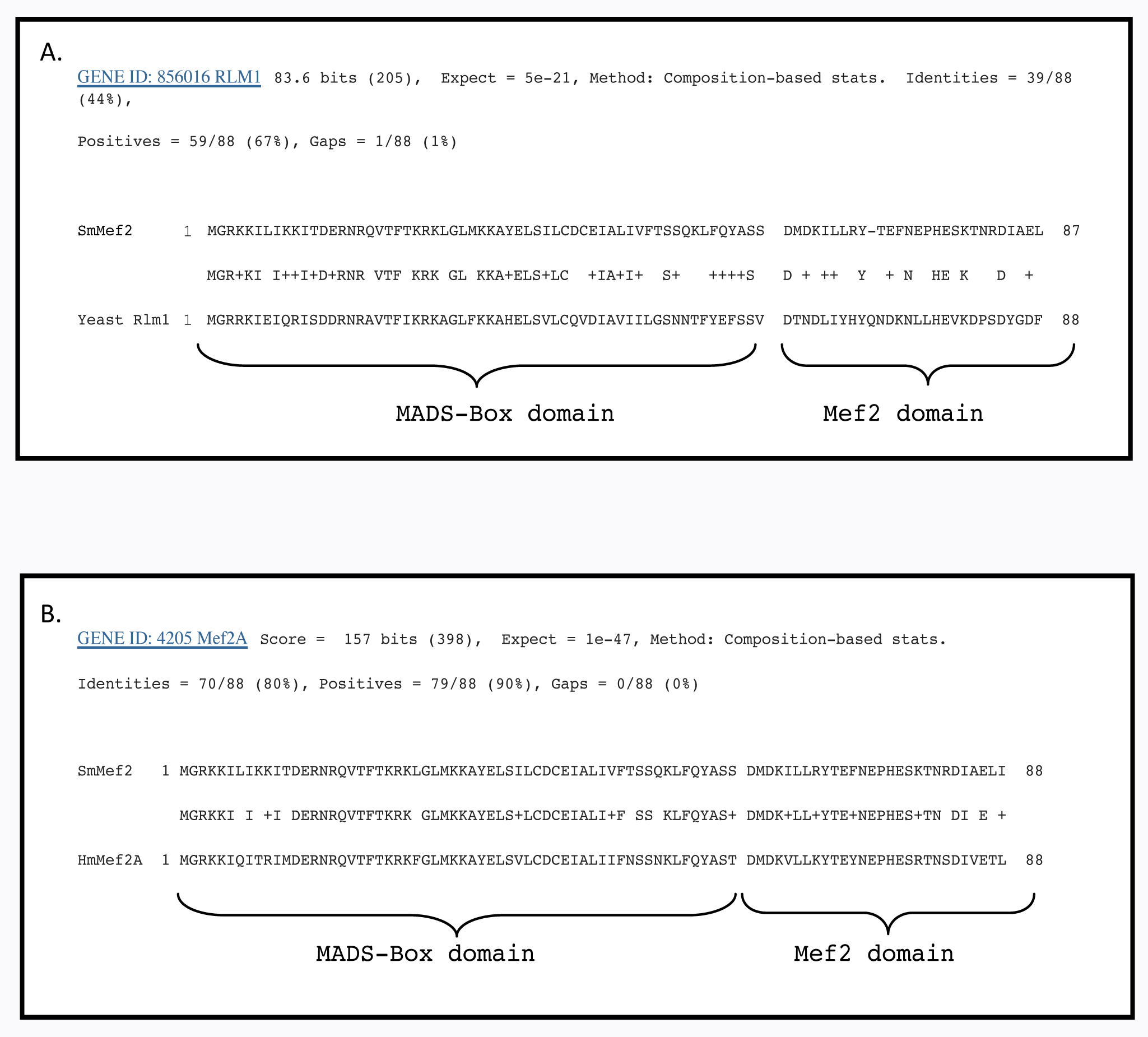

Supplement: Figure S1 — Mef2 Sequence Analysis is seen. Sequence analysis of SmMef2 (Smp_129430) against the yeast gene Rlm1 (A) and human Mef2A (B). The conserved MADS-box (57 amino acids) and the Mef2 domains (29 amino acids) are labeled. (TIF) [file pntd.0001443.s001.tif]

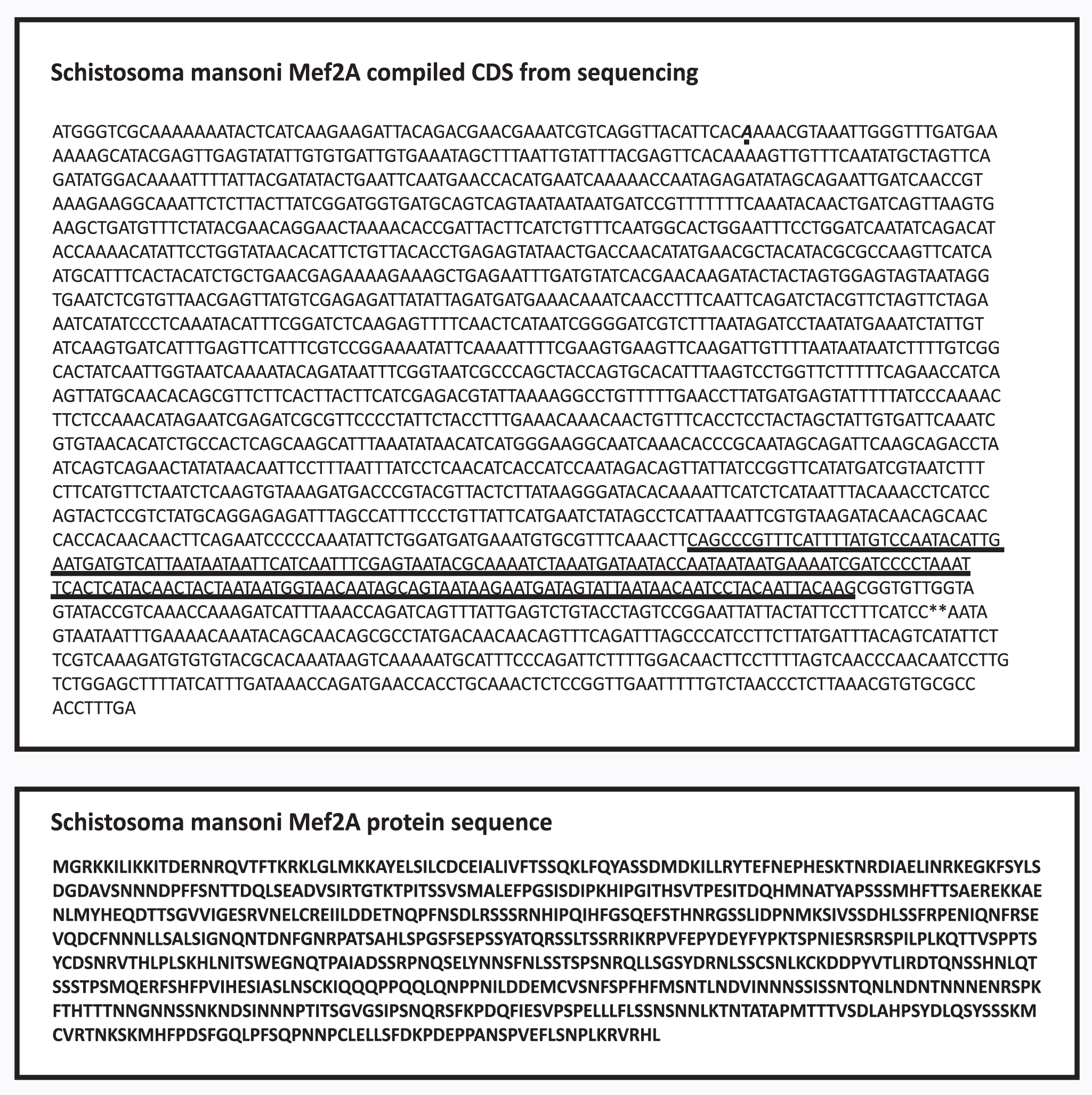

Supplement: Figure S2 — SmMef2 DNA and Protein Sequences are demonstrated. SmMef2 was cloned and sequenced. A G to A transition at nucleotide is underlined, italicized and in bold. An ** is placed after nucleotide 1908 where the deletion of the 10 nucleotides AACAATAAT occurs. The coding sequences between nucleotides 1595 and 1812, previously described as an intron, encode for protein. These sequences are underlined. (TIF) [file pntd.0001443.s002.tif]

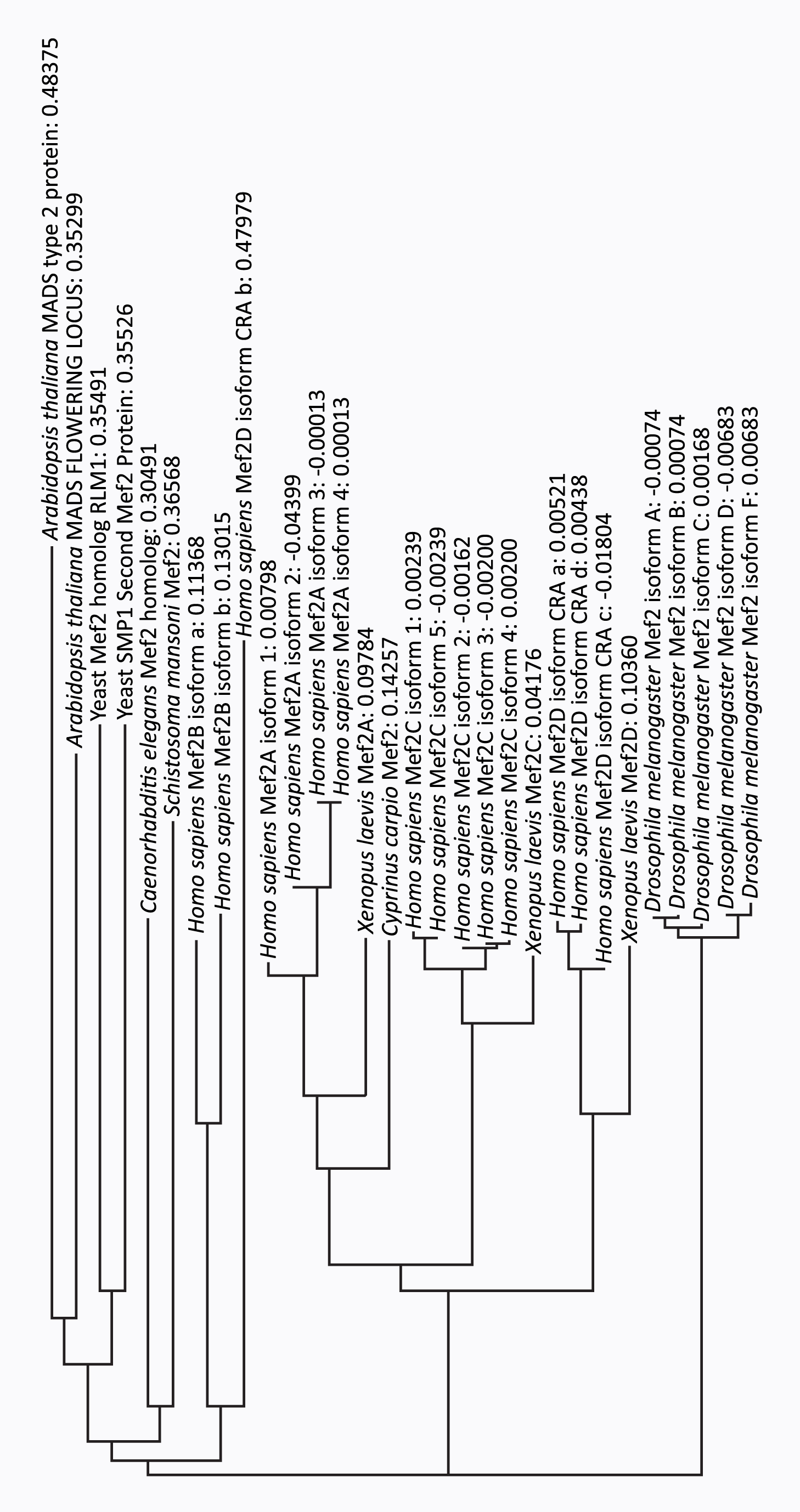

Supplement: Figure S3 — Phylogenetic tree comparison of Mef2 homologs using ClustalW. A phylogram phylogenetic tree was drawn from a ClustalW-generated multiple sequence alignment of Mef2 homologs using the neighbor-joining method (Gonnet protein weight matrix, with the gap open set at 10, the gap extension set at 0.2 and the gap distances set at 5). (TIF) [file pntd.0001443.s003.tif]
