# Supplementary material for: Identification and Characterization of a Mef2 Transcriptional Activator in Schistosome Parasites
Source: PLoS Negl Trop Dis. 2012 Jan 3;6(1):e1443. doi: 10.1371/journal.pntd.0001443 (PMC3250504; doi:10.1371/journal.pntd.0001443)
Supplement: Table S1 — Gene names and DNA primer sequences used for quantitative PCR analysis. (DOC) [file pntd.0001443.s004.doc]

**Table S1. Gene names and DNA primer sequences used for quantitative PCR analysis**

| **Primers for qRT-PCR** | | | |
| --- | --- | --- | --- |
| **Gene name** | **Smp number** | **Forward primer** | **Reverse primer** |
| Cyclophilin | Smp_054330 | TGGGCGGATTTCATAAAGAC | TCGGTTTCAACCCAATTCTC |
| SmMef2 | Smp_129430 | ATATGCTAGTTCAGATATGG | GTAAGAGAATTTGCCTTCTT |
| Tropomyosin | Smp_022170 | TCTCTTACCAAGACTATCAA | CAGTATTTCCATGCGTTTA |
| Netrin | Smp_146840 | GATGTAAACAAGGTGTAGC | TTTATTTGTACTACATGGTGG |
| Tubulin Epsilon Chain | Smp_028360 | ACCAGTGGATCATAAATACT | GACAGTTGTATAATGGTGTAA |
